# Advertising Restrictions and Market Concentration in the Cigarette Industry: A Cross-Country Analysis

**DOI:** 10.3390/ijerph16183364

**Published:** 2019-09-12

**Authors:** Maryam Mirza

**Affiliations:** Institute for Health Research Policy, University of Illinois at Chicago, Chicago, IL 60608, USA; maryamm@uic.edu; Tel.: +1-312-996-1612

**Keywords:** tobacco, health economics, industrial organization

## Abstract

There has been a large increase in the adoption of tobacco advertising restrictions worldwide over the last two decades. Much of the literature studies their direct effect on cigarette demand. This paper investigates the indirect effect of advertising restrictions by evaluating the effect of the policies on the degree of concentration in the tobacco market. By using the variation between countries in timing of adoption of advertising restrictions, I estimate difference-in-difference models to examine the effect of an advertising ban on market-concentration, as measured by HHI. I find that advertising bans lead to an increase in market-concentration: HHI increased by 0.06 points for countries that adopted a ban between 2001 and 2017 conditional on trade and socio-economic characteristics, representing a 13% increase with respect to the mean (0.44). The effect is higher in developing countries (0.08 points increase). Further, I find that ‘comprehensive’ restrictions have a stronger impact on concentration, and ‘limited’ restrictions have little or no impact. These findings point to an important trade-off for policymakers: on one hand, advertising restrictions are likely to reduce consumption of cigarettes; on the other hand, due to an increase in market-concentration, they may be giving more power to tobacco companies.

## 1. Introduction

Tobacco use represents the largest preventable cause of premature death and disease in the world, contributing to over 6 million deaths per year. It is well documented that advertising increases tobacco use; it encourages youth to experiment with tobacco products, reduces current users’ motivation to quit, and encourages former users to resume tobacco use [1]. This link between advertising and smoking is a key concern for policy makers and has led to an extraordinary rise in advertising restrictions across the world. While overall evidence is mixed, prominent studies indicate that advertising restrictions have contributed to a fall in cigarette consumption in high-income countries [2] and developing countries [3]. However, recent theoretical work questions whether this association is causal.

In this paper, I explore the missing link in the studies of advertising restrictions and cigarette consumption. I examine the relationship between tobacco advertising restrictions and market-concentration in the cigarette industry using a cross-country design: a panel dataset for 64 countries covering advertising restrictions for 7 direct media channels, over 17 years, from 2001 to 2017. I use difference-in-difference method to estimate market-concentration, as measured by Herfindahl-Hirschman Index (HHI), in countries that adopted a ban between the years 2001 and 2017 and countries that did not. Employing the same identification strategy, I examine the effect of advertising restrictions on consumption.

Advertising has two equilibrium effects on cigarette demand, the direct effect: it promotes smoking, and the indirect effect: it influences the degree of concentration in the market [4]. Any study on the effect of advertising restrictions must take the effect on concentration into account. Previous papers that have examined indirect effects of advertising restrictions have focused on the 1971 Broadcast Advertising ban introduced in United States. Eckard Jr. [5] provides descriptive evidence that firm concentration was more stable or increasing after the ban. Farr, Tremblay and Tremblay [6] look at price and conclude that there was a fall in market power after the removal of the ban. In contrast to these papers, Bihari and Seldon [7] find a negative effect of advertising ban on market power using Bresnahan and Lerner indices. Together, these papers produce mixed results.

The central question is what is the mechanism through which advertising restrictions may affect market-concentration? I present a theoretical argument based on the premise that changes in market-concentration are driven by changes in a firm’s reputation. Advertising represents an investment that builds up a firm’s stock of goodwill among consumers. It carries over into future periods but loses its value with the passage of time. A ban on advertising increases the cost of firms to reach their consumers, thereby restricting their ability to build goodwill. Firms’ market-shares, then, rely on their past goodwill. In asymmetric market structures typical of the cigarette industry, advertising restrictions disproportionately affect smaller firms (who are at low levels of goodwill to begin with), leading to an increase in market-concentration.

Consistent with the argument summarized above, I find that advertising ban led to an increase in market-concentration: HHI increased by 0.06 points for countries that adopted the ban between 2001 and 2017, conditional on trade and socio-economic characteristics. This estimate represents approximately 13 percent increase with respect to the sample mean (0.44). The effect is higher for developing countries (0.08 points), while developed countries see no effect of an advertising ban. A central concern for these estimates is that advertising restrictions may not be independent events. I explore the dynamics of the ban to test whether, in the absence of adoption of ban, HHI would have trended similarly in countries adopting bans at different times. The pattern of coefficients prior to the ban in developing countries provides strong evidence that adoption of the ban led to HHI increase rather than vice-versa.

A key theme in recent studies of advertising restrictions and cigarette consumption is comparison of varying strengths of policies, based on the idea that if a limited number of mediums are banned, firms can substitute their efforts to reach consumers toward unrestricted media of advertising [2]. I investigate the effects separately for ‘limited’ and ‘comprehensive’ restrictions and find heterogeneity in the effects, that is, limited advertising restrictions have little or no effect on market-concentration in developing countries. Finally, I estimate a simple cigarette demand model to evaluate the effect of advertising restrictions on cigarette consumption considering the indirect effect. Consistent with existing literature, I find that consumption falls after the introduction of advertising bans in developing countries. However, this effect is imprecisely estimated and lower in magnitude.

The results in this paper have important implications for policy. A large body of literature investigates the effect of advertising restrictions on cigarette consumption. These studies, however, are incomplete without consideration of the indirect effect of restrictions through the degree of concentration in the market. My findings extend the literature on indirect effects by providing the first cross-country evidence on the effect of advertising restrictions on market-concentration and incorporating seven direct media channels to make comparisons between varying policy strength. I find that an advertising ban leads to an increase in market-concentration in the cigarette-industry. As concentration rises, prices are likely to rise, potentially leading to a fall in cigarette consumption. This presents an important trade-off for policy makers: efforts to reduce smoking seem to have an unintended effect of increasing market-concentration—giving more power to tobacco companies, which are some of the most powerful and wealthiest companies in existence. 

## 2. Materials and Methods

### 2.1. Data Sources

The main resource for advertising restrictions adopted at country level is the World Health Organizations Tobacco Restriction Policy Data, known as MPOWER [8,9,10] MPOWER contains information on six tobacco control measures identified by WHO FCTC as best practices: Monitor tobacco use and prevention policies, Protect people from tobacco smoke, Offer help to quit tobacco use, Warn people about the dangers of tobacco, Enforce bans on tobacco advertising, Promotion and sponsorship, and Raise taxes on tobacco. Relevant to this study, the dataset provides information on restrictions for the following media: National TV and Radio, International TV and Radio, Local Magazines and Newspapers, International Magazines and Newspapers, Bill Board and Outdoor Advertising, Point of Sale, and Internet, for years 2008, 2010, 2012, 2014, and 2018. Using MPOWER as a benchmark, I supplemented these data by drawing on various other resources, including World Health Organization Regional Office for Europe [11], ERC Group Research Reports, Tobacco Control Laws.org [12], press releases and previous literature. 

Data on market-shares come from Euromonitor Passport Global Market Information Database 2011, 2014, and 2019 editions. Additionally, Euromonitor Passport Database is drawn upon to create controls for summary statistics of the trade environment in each country-year, including cigarette production, imports, and exports. Foreign direct investment (inward flow) is included from United Nations Conference on Trade and Development (UNCTAD) as a percentage of gross domestic product. World Bank World Development Indicators Database was used to create demographic controls such as gross domestic product per capita, population, and unemployment. Finally, to control for other market conditions, smoking prevalence and taxation is sourced from Euromonitor Passport, and prices from Economist Intelligence Unit (EIU). EIU provides four prices for cigarettes: price of a local brand from a low-level market and super-market, and price from Marlboro (or equivalent brand) from the two venues. An average of Marlboro (or equivalent brand) price per pack in each country and year is used. Countries are categorized into income groups based on World Bank’s income level classifications in 2019. High-income countries are classified as developed and middle- and low-income countries are classified as developing countries. The final dataset contains 64 countries and seven media over 17 years, from 2001 to 2017.

### 2.2. Measures

#### 2.2.1. Advertising Restrictions

The adoption of tobacco advertising restrictions varies markedly over time and across countries. Most countries in the sample introduced their first advertising restriction prior to start of the study period, that is, year 2001. The total number of countries having some advertising restriction increased from 39 in 2001 to 61 in 2017. Fifty either made incremental changes to their policy or introduced first restriction during this time. More countries are increasingly moving toward broadly inclusive advertising restrictions. By the end of the period, three countries have yet to introduce any restriction on tobacco advertising: Guatemala, Indonesia, and Japan.

Since developed and developing countries follow different trajectories in terms of tobacco control in general, useful comparisons can be made regarding strength and pace of advertising restrictions between the two groups. Developed, high-income countries tend to have stronger policy restrictions introduced at slow pace; they began early and moved their tobacco control efforts across a wide range of media, over an extended period. Singapore, Poland, Sweden, Finland, Belgium, Italy, Norway and New Zealand have comprehensive set of advertising restrictions prior to 2001, while other countries such as, Germany, UK, Greece and Australia have made incremental changes during period of study. Switzerland, Israel and UK are among the least restrictive countries by 2014. Japan is the only developed country with no advertising restriction on tobacco.

Developing, middle- to low-income countries tend to start their efforts later and have low strength, that is, fewer media restrictions. Lower income countries have widened the scope of advertising restrictions in recent years, even among those that had early starts such as Brazil and Ukraine. Some countries have spurts concentrated in a few years: Costa Rica, Colombia, Ecuador, Philippines and Kenya, while others have made slow consistent efforts through the decade to increase their strength. Peru and Pakistan are trailing, with the slowest start and lowest strength. 

**Figure 1:**
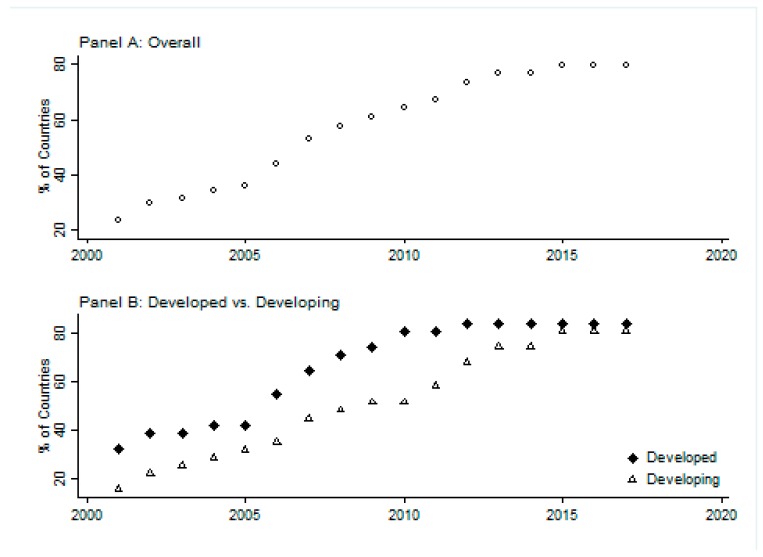
Percent of countries with ‘comprehensive’ advertising restrictions. *Sources:* World Health Organization (WHO)’s Tobacco Restriction Policy Data (MPOWER), WHO Regional Office for Europe, ERC Group Research Reports, Tobacco Control Laws.org, press releases and previous literature.

As noted, there has been a move towards uniformity in tobacco advertising policies across the world: by 2017, most countries have adopted the majority of advertising restrictions regarded by WHO FCTC as best practices. Figure 1 presents a picture of the extent of this uniformity. A policy is classed as comprehensive if five or more media are banned [2]. Panel A plots the percent of countries in the sample that have adopted comprehensive advertising restrictions from 2001 to 2017. Panel B plots developed and developing countries separately. Overall, the basic trend is toward further tobacco control. The total number of countries having a comprehensive set of advertising restrictions increased from 15 in 2001 (23%) to 51 in 2017 (80%). The number is significantly higher in 2017 in both groups, suggesting that advertising control policies have become more restrictive over time.

#### 2.2.2. Market Concentration

Market-concentration is defined as the measure of the extent to which a small number of firms account for a large proportion of an industry’s output. As suggested by Leslie Hannah and John Kay [13], the measure must satisfy a number of generally desirable conditions:

1) An increase in the cumulative share of the i-th firm, for all i ranking firms 1, 2, N in descending order of size, implies an increase in concentration.
2) Switching from smaller to larger firms should increase concentration (Sales Transfer Principle).
3) Entry of new firms, below the average size of existing firms, should reduce concentration (assuming the relative market shares of existing firms remains unchanged).
4) Mergers should increase concentration.
5) If the share of a firm becomes progressively smaller, so should its effect on concentration measure.

In this paper, HHI is used as a measure of market-concentration. It is calculated by aggregating squared-market-shares of all firms. HHI conforms closely to the desirable conditions set above. Specifically, it decreases with the number of firms in the market and increases with variance in market-shares. Moreover, it gives more weight to larger firms. One drawback of HHI, however, is that it requires data for all firms operating in the market. 

Mean HHI in the sample is 0.44. Overall, there is relatively little change in HHI overtime in developed countries; though there is a slight trend toward less concentration. Mean HHI for this region is 0.37. Developing countries, as a whole, are more concentrated, and continue the trend, especially after 2005. Mean HHI for this region is 0.50.

#### 2.2.3. Covariates

Summary statistics for the variables used in the analysis are shown in Table 1. Statistics for developed and developing countries are shown separately.

As mentioned earlier, mean HHI in the sample is 0.44. Developing countries are more concentrated (0.50) than developed countries (0.37). About 84% of the sample is covered under at least one medium of advertising ban. More developed countries have adopted a ban earlier in the period, approximately 93% of this subsample is covered; while developing countries are lagging behind with 74.3% coverage.

There are some prominent differences in other variables. Cigarette prices are more than twice as high in developed countries; while cigarette production is almost three times higher in developing countries. Another noticeable difference is the trade activity: developed countries, on average, have higher exports and imports. Smoking prevalence and foreign direct investment are also higher in developed countries.

### 2.3. Empirical Strategy

The goal of this empirical work is to identify the effect of advertising restrictions on market-concentration. I define ‘restriction’ as a complete advertising ban on at least one medium. Different countries adopted these bans at different times. I exploit this variation in adoption of bans in countries and years to assess the causal relationship between market-concentration and advertising bans. Countries that adopted first ban during the period 2001 to 2017 form the ‘treatment’ group. ‘Control’ group comprises countries that adopted at least one ban prior to 2001 plus countries that have no advertising bans as of 2017. Identifying causal effect requires controlling for any systematic shocks to market-concentration in treatment group that are correlated with, but not due to, the ban.

Specifically, I estimate difference-in-difference models of the form:

(1) $$Y_{jt}=\alpha +\delta ban_{jt}+\mu_{j}+\tau_{t}+\varepsilon_{jt}$$

where the dependent variable is market-concentration measure in country j in year t, and the independent variable is a dummy equal to 1 if a ban is present in country j at year t and 0 otherwise. In addition, the equation includes a vector of country dummies $\mu_{j}$ that controls for mean differences in market-concentration across countries, and year dummies $\tau_{t}$ that control for temporal changes in market-concentration common to all countries. Some models also control for socio-economic characteristics, trade statistics, and cigarette prices. The coefficient of interest is δ, which measures the change in market-concentration due to adoption of the ban.

Ideally, the adoption of a ban will be an independent event that varies in timing and has no spillover effects to other countries. If bans are independent and if no other country characteristic, correlated with change in restrictions, is important to the determination of market-concentration, Equation (1) yields an unbiased estimate of the average treatment effect δ.

#### Potential Threats to Identification

A potential concern for identification is that advertising restrictions may not be independent events. There are two main complications in studying the effect of advertising restrictions on market-concentration. The first complication is the trend toward trade and investment liberalization. ‘Liberalization’ is the term used for removing government restrictions on cross-border trade through bilateral, regional and multilateral trade agreements. Increasing evidence shows that liberalization intensifies competition and promotes consumption [14]. Moreover, low-income economies, which are historically less open, see the largest impact; while high-income economies, which have relatively fewer barriers to trade, see no effect of openness [15]. Similar conclusions have been made regarding foreign direct investment and privatization of state-owned monopolies [16,17,18,19,20]. This trend toward liberalization also has implications for country’s regulatory environment; privatization and higher foreign direct investment increases tobacco industry’s political leverage with respect to tobacco regulation [18,21].

Recent evidence also suggests that tobacco companies consistently use trade and investment agreements to challenge governments’ legislative powers regarding tobacco control measures, through mechanisms such as litigation. The trade agreement directly relevant for the present study is the General Agreement on Trade and Services (GATS), a WTO agreement that extends trade rules to services such as advertising. The GATS requires non-discrimination between imported and domestically produced services; laws may not discriminate in their form (explicitly based on origin) or their effect (disproportionately affect imported services). In addition, GATS restricts countries from maintaining national monopolies, includes limitations on the total value of services supplied and limitation on the total number of service operations [22]. Policy experts claim that General Agreement on Trade and Services (GATS) may be used to challenge advertising restrictions. This is so even if restrictions apply equally to domestic and foreign firms [23]. While there have been a number of disputes involving tobacco products, challenges regarding advertising bans have not come to the WTO. 

The second complication is the socio-economic processes of a country, meaning the conditions that policy makers take into account when recognizing and addressing problems. Factors such as prevalence, overall consumption, economic costs and benefits of tobacco use, and public’s attitude toward smoking form the broader context that tobacco firms and regulators operate in. Regulators are more willing to introduce restrictions if socio-economic conditions are conducive to change. For example, countries with low prevalence and consumption, high health and economic burdens, and low opposition to tobacco regulation are expected to have stronger advertising restrictions. Paul Cairney, Studlar and Maimudi [24] examine the varied policy conditions across regions and find differences between developed and developing countries. Overall, the socio-economic context facilitates tobacco control in developed countries: prevalence, economic value and resistance to tobacco control are generally falling; but it constrains the process in developing countries: economic value of tobacco may be relatively high, prevalence is often rising, and resistance to tobacco control remains strong.

I address these concerns in the following two ways. First, I show results separately for developed and developing countries. Second, I augment Equation (1) with a set of variables relating to trade and socio-economic characteristics of the country, and later add a richer set of covariates based on the discussion above, to check for sensitivity of the baseline results.

## 3. Results

### 3.1. Effect of Advertising Ban on Market-Concentration

Table 2 presents difference-in-difference (DID) estimates of advertising bans and market-concentration as measured by Herfindahl-Hirschman Index (HHI). Model I presents estimates for Equation (1): a regression of HHI on indicator variable for an advertising ban which is equal to 1 if a ban is present in a given country and year, and zero otherwise; Model II and III add controls to address concerns that liberalization, socio-economic context or other time-varying country specific variables are driving the results. Each model contains country and year dummies. Huber-White robust standard errors allow for arbitrary correlation of residuals within each country; all standard errors are clustered at country level.

The first column of Table 2 contains the estimated effect of advertising ban on HHI. The coefficient on ban for Model I indicates that HHI increased by 0.06 points for those countries that adopted the ban between 2001 and 2017 keeping country and year effects constant. Mean HHI in the sample is 0.44. This estimate represents a 14% increase with respect to the mean. A ban on advertising will increase concentration if advertising has become more expensive. In this sense, the principal role of advertising is to create goodwill among consumers and increase their willingness to pay. An advertising ban increases the cost of firms to reach their consumers thereby restricting the ability to build up their goodwill. Firms then operate on the past stock of goodwill, which varies according to the size of the firm, but depreciates over time. Advertising ban disproportionately affects smaller firms, leading to an increase in market concentration.

Above interpretation of the coefficient relies on the assumption that there are no time-varying and country-specific effects correlated with advertising bans. In Model II, I add control variables to address the following concerns. First, that trade and investment liberalization restricts a country’s regulatory freedom to impose advertising bans. Second, that socio-economic context of a country determines the county’s advertising regulation. In Model III, I augment Model II by adding price as an additional control variable. Controlling for price is problematic since price changes may have occurred either because of shifts in demand caused by advertising ban or due to a change in market-concentration. Yet, it is necessary to show whether price-control policies are driving changes in market-share. The results are shown in the second and third column of Table 2.

After controlling for confounding factors, country effects and year effects, the coefficient on ban for Model II decreased by 0.002 points: HHI increased by 0.058 points for the countries that adopted the ban between 2001 and 2017. This estimate represents 12.8% increase with respect to the mean. Finally, in Model III, the coefficient decreases slightly to 0.052 after the addition of price. None of the other variables, apart from imports, has a statistically significant association with HHI in either of the models.

Estimates in Table 2 suggest that adoption of advertising ban results in an increase in market concentration. Given the concern that developed and developing countries follow different trajectories and may experience different impact on market concentration, I can usefully divide the dataset into two groups: developed countries, comprising mature markets with longstanding advertising restrictions; and developing countries, comprising expanding markets with relatively recent advertising restrictions. Table 2 also presents difference-in-difference estimates separately for the two groups. Columns 4-6 show estimates of Model I, II, and III for developed countries and columns 7-9 for developing countries. Table 2 confirms the conclusion that developing countries see an increase in market-concentration after the ban, but shows no effect in developed countries. The estimated impact on HHI is statistically insignificant for developed countries. Not only are the coefficients statistically insignificant, but they are also extremely small. Conditional on confounding variables, the estimated impact of an advertising ban on HHI is 0.01 for Model I and 0.002 for Model II and III.

Developing countries, on the other hand, follow a similar pattern to the pooled sample. The coefficient on ban in column 1 indicates that with constant country and year effects, HHI increased by 0.075 points for those countries that adopted the ban between 2001 and 2017. The impact of controls is higher for this group compared to the pooled sample. After conditioning on confounding variables in Model II (column 8), the coefficient on ban increased slightly: HHI increased by 0.081 points for the countries that adopted the ban between 2001 and 2017. The estimate represents 18 percent increase with respect to mean HHI of the whole sample (0.44), and about 16 percent increase with respect to mean HHI of developing countries (0.5). In Model III (column 9), similar to the pooled sample, controlling for price affects the coefficient minimally: the estimated impact is 0.08 points. None of the other variables, apart from investment is statistically significant in either of the models.

In the following sections, I focus on results from developing countries.

### 3.2. Timing of Adoption and Identifying Assumption

To explore the dynamics of the advertising ban, instead of a simple ban indicator, I use a series of dummy variables indicating time relative to adoption to estimate the effect of advertising ban.

Specifically, I estimate difference-in-difference models of the form:

(2) $$Y_{jt}=\alpha +\overset{10}{\underset{k=-10}{\sum }}\delta ban_{jt,k=t-t_{0}}+\gamma X_{jt}\times \tau_{t}+\tau_{t}+\mu_{t}+\varepsilon_{jt}$$

where the dependent variable is HHI in country *j* in year *t*, and *ban_k,jt_* is a dummy variable equal to 1 if a country is *k* years relative to the adoption of a ban and zero otherwise (For example, if ban is introduced in a country in 2014, *k* will take the value of 0 in 2014, -1 in 2013, and 1 in 2015; *ban* will be 1 for years where *k* is equal to or greater than 0, that is 2014.) In addition, the equation includes a vector of country dummies, *µ_j_*, that controls for mean differences in market-concentration across countries; year dummies, *τ_t_*, that controls for temporal changes in market-concentration common to all countries; and trade and socio-economic variables interacted with time dummies to control for any additional variation that may be driving the results, *X_jt_ × τ_t_*. The coefficients *δ_k_* for *k >* 0, measure the change in market-concentration due to adoption of the ban.

An advantage of this model is that it allows for partial test of the identifying assumption, that is, in the absence of adoption of an advertising ban, HHI would have trended similarly in countries adopting bans at different times. If the timing of adoption is unrelated to underlying trends and tobacco industries within countries do not respond before adoption, there should be no trend in the *δ_k_* for *k ≤* 0.

Taken as a whole, the pattern of *δ_k_*’s describes the change in trend in HHI associated with adoption of advertising bans. Estimates are found in Table 3 Panel A. Note that the indicator for year 10 is equal to 1 for each subsequent year, starting with year 10; similarly the indicator for year 10 prior to the ban is equal to 1 for year 10 and prior years. Note also that since countries adopt bans in different years, the composition of countries identifying *δ_k_* varies with *k*. In the presence of heterogeneity, the pattern of the *δ_k_*’s reflects changes in the composition of countries that identify the coefficient as well as the dynamics of the effect of advertising ban. For example, if effect of the ban is higher for countries that have adopted the ban later in the study period, the *δ_k_*’s for later years will be smaller than the years closer to the ban. Similarly, estimates of pre-trend may also be affected by compositional changes. This does not represent a violation of the identifying assumption; it is interpreted as a heterogeneous treatment effect. Finally, note that the omitted category is the year before adoption of ban.

The coefficients on ban leads in Table 3 Panel A, six and more years prior to adoption range between 0.16 to 0.05; however, the standard errors are very high for these estimates. Lack of information, since few countries with six or more pre-ban years, and compositional changes are most likely driving these estimates. The coefficient of ban leads three to four years prior to ban are close to zero, showing little evidence of an anticipatory response within developing countries about to adopt an advertising ban. In the second year of adoption, HHI increases slightly; in the third year, HHI increases substantially by 0.07 points, after which the coefficients fluctuate between 0.07 to 0.09 points till year eight; the coefficient then averages 0.07 points in subsequent years. This pattern of coefficients prior to the ban provides evidence that adoption of the ban led to HHI increase rather than vice-versa in developing countries.

Next, I estimate the level and trend in HHI before the adoption of ban, and changes in level and trend in HHI, after the adoption of ban. Specifically, I estimate regression model of the form:

(3) $$Y_{jt}=\alpha +\delta ban_{jt}+\gamma T_{t}+\gamma^{\prime }{T^{\prime }}_{t}+\mu_{j}+\varepsilon_{jt}$$

where the dependent variable is HHI in country *j* in year *t*, and the independent variable is a dummy equal to 1 if a ban is present in country j at year t and 0 otherwise. A yearly variable, *T,* is the trend before the adoption of ban, and *T^t^* is the trend after the ban. In addition, the equation includes a vector of country dummies, *µ_j_*, which controls for mean differences in market-concentration across countries. The coefficient, *δ*, measures the change in the level of market concentration due to adoption of the ban; it represents the immediate effect of the ban on HHI. The coefficient, *γ*, measures the average change in HHI per year; it denotes the underlying trend. The coefficient, *γ*’, measures the change in trend after the adoption of the ban; it is interpreted as the gradual change in the underlying trend of HHI.

Estimates of this regression are found in Panel B of Table 3. Overall, coefficients on trend variables, *T* and *T’*, suggest that there is neither an underlying trend nor a change in trend following the adoption of the ban. The main impact is a change in the level of market-concentration. The coefficient on ban indicates that after removing mean country HHI, and before and after trends, HHI increased by 0.08. This coefficient is directly comparable to Table 2, column 7, which contains a coefficient of 0.07, providing further support for the empirical strategy.

### 3.3. Level of Regulation

The results so far suggest that the introduction of a tobacco-advertising ban leads to an increase in market-concentration in the cigarette industry in developing countries and has no effect on concentration in developed countries. However, they do not make a distinction regarding which medium of advertising is banned, or how many media are banned.

Previous literature on the effect of advertising bans on consumption indicates the importance of looking at the effect of ‘comprehensive’ advertising restrictions [2]. Merely having any ban in the country will not have a significant impact, as firms can substitute their efforts toward the unrestricted medium of advertising. Several alternatives have been used to classify restrictions such as: (i) a bounded score from 0 to 10 [25]; (ii) dummy variables for warning requirements, tv & radio bans, moderate bans (4 media banned), and strong bans (5 or more) [26]; and iii) dummy variables for weak (0-2), limited (3-4) and comprehensive (5 or more) [2,3]. While these authors provide useful ways to characterize comprehensive bans, there is still some ambiguity in literature regarding how to measure, classify, and interpret the level of restrictions mainly due to the subjective nature of these classifications.

To examine the effect of the level of restrictions I follow Saffer and Chaloupka’s [2] approach and introduce three new variables designed to measure weak, limited and comprehensive regulations. Each set is defined in two ways. The first definition is based on the idea that larger number of media bans leads to a more comprehensive restrictions. Policies are assigned as weak if there is no complete ban on any media, or have partial bans on any media; limited if one to three media are banned; and comprehensive if four or more media are banned. The second definition uses specific media bans to define policies. While the definition of a weak policy is the same, policies are assigned as limited if either tv & radio are banned, or tv and radio as well as billboards are banned; and comprehensive if tv and radio, billboards and any other media are banned.

This classification, as slightly differs from existing literature, is motivated by three considerations. First, tv and radio are traditionally the most important media for tobacco advertising. They are always combined together, especially after year 2000, and are also the first media to be restricted or banned. To be precise, if there is only one media banned in a country, it is almost always tv & radio. Assigning a weak status to ban on tv and radio misrepresents the strength of the policy. Second, previous literature includes sponsorships, an indirect form of advertising, in their analysis. The present study is focused on direct forms of advertising. Third, broadly, the classification into different levels of policy is based on evidence, from journal papers, newspaper articles, etc., on the importance of advertising media to consumption.

The level of restrictions or strength of policy can be studied within the same regression framework described in Section 2.3. In this section, I substitute ban dummy with two dummies, limited and comprehensive, indicating the level of restrictions present in a country and year. Estimates are found in Table 4. Model I presents estimates for Equation (1); Model II adds controls for trade and socio-economic characteristics; and Model III adds price. In both these models, I add an additional control variable measuring years since first ban. As discussed in Section 2, countries take different course to stronger restrictions: some countries make incremental changes over an extended period, others have spurts concentrated in a few years. The variable is meant to account for different impacts for countries that switch policy from limited to comprehensive and countries that switch from weak/no ban to comprehensive. Panel A presents results using the first definition for weak, limited and comprehensive policies; Panel B presents the second. The dependent variable in this table is HHI. Robust standard errors are shown in parentheses, clustered at country level.

Consistent with recent literature on the effects of advertising restrictions on consumption [2,3]. Panel A shows that having a more comprehensive level of regulation leads to larger effect on the market. After controlling for socio-economic characteristics and price, the coefficient shows that the impact of limited and comprehensive policies is similar. Model III yields an estimate of 0.081 for limited policies and 0.095 for comprehensive policies. 

Panel B uses the second definition for strength of policy. The most prominent differences between the two panels is that the coefficient on limited is slightly lower in panel B, that is, when defined with specific media. This indicates that a ban only on tv and radio and billboards have a smaller effect on HHI. The coefficients on comprehensive are positive and statistically significant, and are similar in magnitude compared to panel A. The introduction of limited policies increases HHI by 0.07 points, whereas comprehensive policies will increase HHI by 0.1 points, conditional on other covariates.

Table 4 reveals that strength of advertising restriction correlates with an increase in market-concentration, as measured by HHI. Whether the strength is defined using the first or second method, coefficients on comprehensive dummies are statistically significant and positive. The coefficients on limited, however, are dependent on the classification. The sensitivity of the coefficients to different classifications is problematic, and suggests the necessity of finding a consistent method to define strength of policies.

### 3.4. Effect on Consumption

In this section, I apply the same empirical strategy to estimate the effect of advertising restrictions on consumption. Specifically, I estimate difference-in-difference models of the form:

(4) $$Y_{jt}=\alpha +\delta ban_{jt}+\mu_{j}+\tau_{t}+\varepsilon_{jt}$$

where the dependent variable is logged consumption per capita in country *j* in year *t*, and the independent variable is a dummy equal to 1 if a ban is present in country *j* at year *t* and 0 otherwise. In addition, the equation includes a vector of country dummies, $\mu_{j}$, that controls for mean differences in market-concentration across countries, and year dummies, $\tau_{t}$, that control for temporal changes in market-concentration common to all countries. Some models also control for socio-economic characteristics, market-concentration, and cigarette prices. The coefficient of interest is *δ*, which measures the change in consumption due to adoption of the ban.

Table 5 presents the results. Model I presents estimates for Equation (4), that is, a regression of logged consumption per capita on indicator variable for an advertising ban which is equal to 1 if a ban is present in a given country and year, and zero otherwise; Model II adds controls for price, unemployment, percent of female population, and an indicator measuring the number of years since advertising first ban was introduced in the country; Model III adds HHI. Each model is estimated for all, developed, and developing countries separately. Huber-White robust standard errors allow for arbitrary correlation of residuals within each country; all standard errors are clustered at country level.

None of the models estimated in this table produce a statistically significant association between log of consumption per capita and advertising ban. In Panel A, the coefficient on ban for Model I indicates that after removing mean country consumption per capita and common year effects, logged consumption per capita decreased by 0.06 points for those countries that adopted the ban between 2001 and 2017. After controlling for confounding factors, country effects and year effects, the coefficient on ban for Model II decreased to 0.07 points. Finally, in Model III, the coefficient increases to 0.06 after the addition of HHI. 

I next examine the effect of the level of restriction on consumption. Table 5 Panel B presents the estimates for limited and comprehensive restrictions. Similar to Table 4, Table 5 presents two alternative classifications. The results for both classifications are qualitatively similar. After controlling for confounding variables, the direction of the effect is negative for limited as well as comprehensive restrictions. Limited restrictions have smaller effect on consumption than comprehensive restrictions. However, these estimates are imprecisely estimated.

## 4. Discussion

The literature identifies a contrast between developed and developing countries. In the former, policy changes have taken place over an extended period of time driven by domestic institutions, efforts of public health groups, scientific knowledge and changing social attitudes and behaviors. In the latter, few domestically driven policy measures are apparent and domestic public health capacity is relatively low; policy has developed through policy transfer from developed countries led by institutions such as WHO, World Bank, and the UN, the most prominent contribution being the formation of the 2003 Framework Convention on Tobacco Control (FCTC). Moreover, trade environment and its impact on market is markedly different between the two regions. Consequently, a study focused on all countries grouped together may have limited value. This paper identifies a contrast between developed and developing countries: advertising restrictions have a positive impact on market-concentration in developing countries but have no statistically significant in developed countries. 

The absence of an effect in developed countries is not surprising. As the discussion of adoption of advertising restrictions in Section 2.1 demonstrates, many developed countries had already introduced a range of bans prior to 2001. Evidence also indicates that socio-economic context is more receptive to tobacco control in these countries, which has led to organized, broadly inclusive policies aimed at reducing tobacco use. As a result, smoking prevalence is on a decline. To state more formally, developed countries are at later stages of the ‘tobacco epidemic model’ as proposed by Lopez, Collishaw, and Piha [27]: knowledge of smoking risks is widespread, effective systems to curb tobacco use are in place, and tobacco control has had a substantial success, resulting in falling smoking prevalence. In fact, the experience of most developed countries serves as a learning tool for other countries to intervene in earlier stages. In this light, the absence of an effect of an advertising ban on concentration, which is essentially driven by consumers’ willing to pay, is not alarming.

On the other hand, the magnitude of the effect in developing countries is important. Developing countries are at different stages of the tobacco epidemic. Sub-Saharan African countries have low smoking rates and have not experienced the harmful effects of smoking; while Latin American and Caribbean countries, and Middle East and North African countries have started to experience the harmful effects [24,28]. Overall, smoking prevalence in developing countries is rising, which underpins the significance of effective advertising restrictions as a means of early intervention. The results in this paper show that an advertising ban leads to an increase in market concentration. Economic theory suggests that an increase in concentration is most likely to increase price, reinforcing the direct effect on cigarette demand, leading to a negative impact on consumption-the indicated objective of the ban. However, there is an irony to these findings. Higher concentration in the cigarette industry implies an increase in market power of transnational tobacco companies. These companies are some of the most powerful and richest companies in the world, richer than most developing countries. It is also widely documented that the presence and market-power of transnational tobacco companies is associated with an increase in smoking prevalence, especially among women [16,18,20,29]. In this light, the magnitude of the effect of an advertising ban on concentration presents an important trade-off for policymakers: falling consumption may mean compromising on market power for tobacco companies.

Section 3.4 studies the impact of advertising restrictions after accounting for both, direct and indirect effects of market-concentration. However, none of the models estimated produce a statistically significant association between log of consumption per capita and advertising ban. This effect of advertising restrictions on consumption differs from previous literature. In the most recent work on this topic, Blecher [3] concludes that restrictions lead to a 23 percentage point fall in consumption per capita in developing countries. There are two major differences between Blecher’s study and the present analysis. First, dataset in this paper is smaller and recent. An explanation for the difference in estimated effect may be diminishing returns to restrictions. As more restrictions are introduced and socio-economic context becomes more receptive to tobacco control, the effect of further restrictions becomes inconsequential. The second difference is the composition of countries, which possibly leading to different effect sizes. 

Note that this analysis has focused solely on direct forms of advertising. Indirect forms of advertising, such as sponsorships, are also important channels through which firms reach their consumers. The question of interest is how important are these channels for market-concentration? Do they serve as substitutes or complements? Additionally, in studying the level of restriction, I have attempted to provide an overall sense of interaction between strength and concentration; however, the estimates are sensitive to different classifications. I can potentially combine the insights of literature on media-penetration to create a country-specific index weighted by the relative importance of a particular media. Finally, this paper concentrated on estimating whether a tobacco advertising ban has an effect on market-concentration, and the dynamics and strength of the ban. Another interesting question to study is whether the advertising ban changes the number of firms in the market, or affect domestic firms in favor of foreign firms.

## 5. Conclusions

By using the variation between countries in timing of adoption of advertising restrictions, this paper estimates the effect of an advertising ban on market-concentration, measured by Herfindhal-Hirschman Index (HHI). An advertising ban makes it more expensive for firms to reach their consumers and build up their goodwill. Smaller firms, who have low goodwill to start with, are disproportionately affected by the ban, which may lead to an increase in market-concentration. In addition, I examine whether the effect varies with the level of restrictions. Specifically, do comprehensive advertising restrictions have a stronger effect than limited advertising restrictions.

The evidence provides support for a positive relationship between advertising restrictions and market-concentration. The estimates of advertising ban on HHI in Section 3.1 suggest that HHI increased by 0.06 points for countries that adopted the ban between 2001 and 2017, after removing mean country and common year effects, and conditional on trade and socio-economic characteristics. This estimate represents approximately 13% increase with respect to the sample mean (0.44). In developing countries, the comparable model estimates a coefficient of 0.081, representing an increase of 18% with respect to mean HHI of the whole sample (0.44), and about 16% increase with respect to mean HHI of developing countries (0.5). In developed countries, on the other hand, an advertising ban does not have an effect on concentration. In addition, I find that comprehensive restrictions have a stronger effect on concentration while limited restrictions have little or no effect.

Estimates of the effect of advertising restrictions on cigarette consumption are less conclusive. In Section 3.4, I find that consumption per capita falls after the adoption of bans in developing companies. However, the estimates lack statistical significance.

The findings revealed in this paper have important implications for policy. First, they provide a key piece of information regarding the effect of advertising restrictions on consumption—the indirect effect of restrictions through the degree of concentration in the market. Second, they present as an important trade-off for policymakers: on one hand, advertising restrictions are likely to reduce consumption of cigarettes; on the other hand, due to an increase in market concentration, they may be giving more power to tobacco companies. Third, they inform the discussion on tobacco policy in developing countries. Despite a major change in policies, there still exists a large policy divide between developed and developing countries. Moreover, per capita rate of consumption in developed countries, though higher, is falling; but in developing countries, the rate is rising and expected to rise even further in the future. This is particularly important because by 2030, eighty percent of tobacco related deaths might occur in developing countries.

## Figures and Tables

**Table 1:** Summary Statistics.

	All	Developed	Developing
	(*n =* 1088)	(*n =* 544)	(*n =* 544)
HHI	0.44 (0.2)	0.37 (0.2)	0.50 (0.21)
Ban (%)	83.5 (37.0)	92.83 (25.8)	74.3 (44.8)
Price (USD)	3.92 (3.0)	5.76 (3.3)	2.07 (1.0)
GDP Per Capita (000, Constant 2010 USD)	21.56 (21.1)	37.99 (18.5)	5.14 (3.2)
Foreign Direct Investment, Inward *(*% *of GDP)*	3.23 (4.6)	3.67 (5.6)	2.86 (3.5)
Population, Female (%)	50.1 (3.1)	49.99 (4.2)	50.4 (1.2)
Population, 65+ (%)	10.91 (5.7)	14.85 (4.7)	6.98 (3.5)
Unemployment, Total (%)	7.75 (5.0)	7.74 (4.2)	7.76 (5.6)
Smoking prevalence, Female (%)	14.53 (8.6)	19.70 (6.6)	9.37 (7.3)
Smoking prevalence, Total (%)	23.69 (7.3)	24.77 (6.6)	22.61 (8.0)
Production (Bn)	83.28 (280.8)	43.68 (84.2)	122.89 (384.2)
Exports (Bn)	13.12 (26.6)	19.98 (35.0)	6.30 (10.2)
Imports (Bn)	10.04 (17.6)	17.13 (22.4)	2.95 (4.7)

Sources: Euromonitor International Limited, United Nations Conference on Trade and Development (UNCTAD), Economist Intelligence Unit (EIU), World Health Organization (WHO), and World Bank.

**Table 2:** Estimated impact of advertising ban on market concentration.

	All			Developed Countries			Developing Countries		
	I	II	III	I	II	III	I	II	III
	(1)	(2)	(3)	(4)	(5)	(6)	(7)	(8)	(9)
Ban	0.061 **	0.059 **	0.052 **	−0.011	0.002	−0.002	0.075 **	0.081 ***	0.080 ***
	(0.025)	(0.025)	(0.024)	(0.052)	(0.054)	(0.051)	(0.030)	(0.030)	(0.027)
GDP per capita (Constant 2005 US$)		−0.002	−0.000		0.008	0.001		−0.015	−0.010
		(0.002)	(0.002)		(0.003)	(0.003)		(0.012)	(0.012)
Population, Female (% of total)		0.002	−0.003		−0.012	−0.012		0.020	0.029
		(0.018)	(0.018)		(0.022)	(0.022)		(0.092)	(0.086)
Unemployment, Total		−0.003	−0.002		−0.002	−0.001		−0.004	−0.003
		(0.002)	(0.002)		(0.003)	(0.002)		(0.005)	(0.005)
Smoking Prevalence		0.0702	0.017		0.282	0.251		−0.337	-0.369
		(0.242)	(0.235)		(0.326)	(0.329)		(0.308)	(0.305)
FDI, Inward (Percent of GDP)		−0.000	−0.000		0.000	0.000		−0.002 **	−0.002 **
		(0.001)	(0.001)		(0.000)	(0.000)		(0.000)	(0.001
Production		0.000	−0.000		−0.000	−0.000		0.000	0.000
		(0.000)	(0.000)		(0.000)	(0.000)		(0.000)	(0.000)
Exports		0.000	0.000		0.000	0.000		0.001	0.002
		(0.000)	(0.000)		(0.001)	(0.001)		(0.001)	(0.001
Imports		−0.001 *	−0.001 *		−0.001	−0.001		−0.003	−0.003
		(0.000)	(0.001)		(0.000)	(0.000)		(0.002)	(0.002)
Price			−0.008 **			−0.003			−0.034 **
			(0.003)			(0.003)			(0.0151)
R^2^	0.910	0.912	0.913	0.918	0.921	0.921	0.896	0.901	0.906

Notes: All models include country fixed effects and year fixed effects. *N (All)* = 1076, *N(Develop)* = 544, *N(Developing)* = 532. Standard Errors are clustered by country. * significant at 10%; ** significant at 5%; *** significant at 1%.

**Table 3:** The dynamics of the effect of advertising restrictions in developing countries.

Panel A		Panel B	
Ban_t-10 or more_	−0.148 *	Ban	0.084 ***
	(0.083)		(0.030)
Ban_t-9_	−0.161 *	Slope before Ban	−0.0021
	(0.089)		(0.002)
Ban_t-8_	−0.142 *	Slope after Ban	0.004
	(0.077)		(0.004)
Ban_t-7_	−0.102		
	(0.067)		
Ban_t-6_	−0.071		
	(0.057)		
Ban_t-5_	0.059		
	(0.038)		
Ban_t-4_	−0.049		
	(0.031)		
Ban_t-3_	−0.009		
	(0.023)		
Ban_t-2_	0.008		
	(0.013)		
Ban_t_	0.005		
	(0.013)		
Ban_t+1_	0.006		
	(0.010)		
Ban_t+2_	0.043		
	(0.031)		
Ban_t+3_	0.067 **		
	(0.032)		
Ban_t+4_	0.065		
	(0.040)		
Ban_t+5_	0.086 **		
	(0.038)		
Ban_t+6_	0.108 ***		
	(0.039)		
Ban_t+7_	0.070		
	(0.049)		
Ban_t+8_	0.091 *		
	(0.045)		
Ban_t+9_	0.076		
	(0.048)		
Ban_t+10_	0.093		
	(0.064)		
R^2^	0.921		

Notes: All models include country fixed effects. *N* = 544. Standard Errors are clustered by country. * significant at 10%; ** significant at 5%; *** significant at 1%.

**Table 4:** The estimated impact of level of restrictions on market concentration.

	Panel A			Panel B		
	I	II	III	I	II	III
Limited	0.094 ***	0.085 ***	0.081 ***	0.068 **	0.074 **	0.069 **
	(0.032)	(0.025)	(0.025)	(0.032)	(0.031)	(0.029)
Comprehensive	0.097 ***	0.098 ***	0.095 ***	0.101 ***	0.010 ***	0.099 ***
	(0.033)	(0.032)	(0.029)	(0.032)	(0.030)	(0.027)
R^2^	0.87	0.88	0.88	0.87	0.88	0.89

Notes: Panel A: Limited (1–3), Comprehensive (4+) Panel B: Limited (tv and radio are banned, or tv and radio as well as billboards are banned), Comprehensive (tv and radio, billboards and any other media are banned). All models include country fixed effects and year fixed effects. *N* = 544. Standard Errors are clustered by country. * significant at 10%; ** significant at 5%; *** significant at 1%.

**Table 5:** The estimated impact of level of restrictions on market concentration.

	**Panel A**					
	**I**		**II**		**III**	
Ban	−0.055		−0.067		−0.061	
	(0.070)		(0.065)		(0.061)	
R^2^	0.98		0.98		0.98	
	**Panel B**			**Panel C**		
	**I**	**II**	**III**	**I**	**II**	**III**
Limited	−0.036	−0.045	−0.043	−0.006	−0.007	0.009
	(0.090)	(0.070)	(0.070)	(0.082)	(0.077)	(0.075)
Comprehensive	−0.063	−0.098	−0.097	−0.067	−0.078	−0.074
	(0.074)	(0.059)	(0.057)	(0.073)	(0.061)	(0.074)
R^2^	0.98	0.98	0.98	0.98	0.98	0.98

Notes: All models include country fixed effects and year fixed effects. *N* = 544. Standard Errors are clustered by country. * significant at 10%; ** significant at 5%; *** significant at 1%.
